# Serum Uric Acid as a Biomarker of Disease Severity in Children With Complicated Malaria: A Cross-Sectional Study From Eastern India

**DOI:** 10.7759/cureus.106414

**Published:** 2026-04-03

**Authors:** Mukesh Kumar, Sunanda Jha, Hansraj Kumar, Barapatla Sarangam, Priya K Munda, Varun Garg, Shiv K Mudgal

**Affiliations:** 1 Pediatrics, Phulo Jhano Medical College and Hospital, Dumka, IND; 2 Pediatrics, Rajendra Institute of Medical Sciences, Ranchi, IND; 3 Pharmacology and Therapeutics, All India Institute of Medical Sciences Deoghar, Deoghar, IND; 4 Pediatrics and Neonatology, Mahatma Gandhi Memorial Medical College and Hospital, Jamshedpur, IND; 5 General Medicine, Rajendra Institute of Medical Sciences, Ranchi, IND; 6 Pediatrics, Rajendra Institute of Medical Sciences,Ranchi, Ranchi, IND; 7 College of Nursing, All India Institute of Medical Sciences Deoghar, Deoghar, IND

**Keywords:** biomarkers, hyperuricemia, pediatric malaria, plasmodium falciparum, severe malaria

## Abstract

Background: Malaria continues to be a significant contributor to morbidity among children in tropical countries. Increasing evidence suggests that elevated uric acid levels may reflect inflammatory activity and disease severity in malaria. The aim of this study was to measure serum uric acid (SUA) levels in children with complicated malaria and to examine their association with clinical outcomes and biochemical markers of hepatic dysfunction.

Methods: A hospital-based cross-sectional investigation was carried out in the Pediatrics Department of a tertiary care teaching hospital in eastern India from 01/09/2022 to 31/05/2024. A total of 102 children aged 1-18 years with laboratory-confirmed complicated *Plasmodium falciparum* malaria were enrolled using purposive sampling. Clinical details and laboratory parameters, which include SUA, serum glutamic oxaloacetic transaminase (SGOT), serum glutamic pyruvic transaminase (SGPT), total bilirubin, and C-reactive protein (CRP), were recorded. IBM SPSS Statistics for Windows, Version 23 (Released 2015; IBM Corp., Armonk, New York, United States) was used for statistical analysis.

Results: Hyperuricemia was detected in 80.4% (82) of the participants. Elevated SGOT, SGPT, and bilirubin levels were observed in 72.5% (74), 83.3% (85), and 65.7% (67) of cases, respectively. Adverse outcomes occurred in 29.4% (30) of patients. Significant associations were found between adverse outcomes and abnormal SGOT (χ²=6.499, p=0.011), SGPT (χ²=8.500, p=0.004), and bilirubin (χ²=8.300, p=0.004), whereas SUA levels were not significantly associated with outcomes (p=0.115).

Conclusion: Hyperuricemia is common in pediatric complicated malaria and may reflect infection-related inflammation. However, SUA alone may not reliably predict adverse clinical outcomes. Larger multicenter studies are needed.

## Introduction

Malaria continues to be among the most important parasitic infections worldwide and continues to be a serious public health issue. According to recent global reports, hundreds of millions of malaria cases are recorded each year, with children living in endemic regions bearing a large proportion of severe illness and deaths associated with the disease [1]. Although substantial progress has been achieved in the prevention and management of malaria through vector management strategies, chemoprophylaxis, and improved diagnostic techniques, severe malaria continues to contribute significantly to pediatric morbidity and hospitalization in many countries that are tropical or subtropical [2,3].

Within the Southeast Asian region, India still accounts for a considerable share of malaria cases. Despite the implementation of national malaria elimination programs that have reduced transmission in several regions, certain states such as Jharkhand remain endemic. Persistent transmission in these areas is influenced by environmental conditions that are conducive to mosquito growth, socioeconomic inequalities, and limitations in healthcare accessibility [4,5]. Children residing in these settings are particularly vulnerable because of their incomplete immunity to malaria parasites and greater susceptibility to severe forms of infection.

Among the malaria parasites infecting humans, *Plasmodium falciparum* is responsible for most cases of severe disease and malaria-related mortality worldwide [6]. Severe malaria results from complex interactions between parasite multiplication and host immune responses, often leading to dysfunction of multiple organ systems. Common clinical manifestations include respiratory distress, metabolic acidosis, severe anemia, cerebral malaria, and hepatic impairment [7-9]. These complications arise due to several pathological mechanisms such as sequestration of infected erythrocytes in microvasculature, destruction of red blood cells, endothelial activation, and systemic inflammatory responses induced by parasite-derived factors [10].

Inflammatory processes play a vital role in the pathogenesis of malaria. During the erythrocytic stage of infection, rupture of parasitized red blood cells releases parasite antigens and metabolic products into the circulation. These substances activate innate immune pathways and stimulate the release of pro-inflammatory cytokines, including tumor necrosis factor-α, interleukin-1β, and interleukin-6 [11-13]. While these immune responses contribute to parasite clearance, excessive inflammatory activation may result in endothelial injury, tissue damage, and ultimately organ failure in severe malaria [14].

Recently, uric acid has been identified as a potential mediator involved in malaria-related inflammation. During the intra-erythrocytic developmental stage of *Plasmodium*, infected red blood cells accumulate significant quantities of hypoxanthine, a precursor molecule in purine metabolism. When infected erythrocytes rupture, hypoxanthine is released into the bloodstream and subsequently converted into uric acid by the enzyme xanthine oxidase [15,16]. There is growing evidence that uric acid may function as a danger-associated molecular signal capable of activating immune cells and enhancing inflammatory responses [17].

Experimental studies have indicated that uric acid can stimulate dendritic cells and macrophages through activation of inflammasome pathways, thereby promoting the release of cytokines that cause inflammation, which contributes to systemic inflammation [18]. In malaria infection, elevated levels of uric acid may therefore participate in endothelial activation and microvascular dysfunction, which are important pathogenic characteristics of severe disease [19].

Many recent clinical studies have reported increased amounts of plasma uric acid in malaria patients, particularly in patients with severe infection. Lopera-Mesa and colleagues observed that uric acid concentrations were associated with markers of inflammation and endothelial activation in pediatric malaria cases [20]. Other investigations have also indicated that hyperuricemia may correlate with parasite density and severity of illness [21-23].

Despite these findings, the clinical significance of elevated uric acid levels in malaria is still not clearly defined. In particular, limited information is available regarding the relationship between serum uric acid (SUA) concentrations and disease severity among children living in malaria-endemic areas. Identification of reliable biochemical markers may assist clinicians in early detection of severe disease and improved risk stratification. Therefore, the current study was carried out to determine SUA levels in children with complicated malaria admitted to a tertiary care hospital in eastern India and to assess their association with disease severity and laboratory indicators of organ dysfunction.

## Materials and methods

Study design, setting, and duration

The present investigation was designed as a hospital-based cross-sectional investigation carried out in the Pediatrics Department of Rajendra Institute of Medical Sciences, Ranchi, India. Data collection was done from 01/09/22 to 31/05/2024 in accordance with the predefined study protocol.

Study population

The study included children aged between 1 and 18 years who were admitted to the pediatric department with laboratory-confirmed *Plasmodium falciparum* malaria and clinical features suggestive of complicated malaria.

Inclusion and exclusion criteria

Children aged 1-18 years diagnosed with *Plasmodium falciparum* infection and meeting the requirements for severe malaria set out by the World Health Organization (WHO) were considered eligible for inclusion in the study [24]. Patients were excluded if they had any known medical conditions associated with increased uric acid levels that could potentially influence SUA measurements. In addition, patients were not included if informed consent from parents or legal guardians was not obtained prior to enrollment.

Sampling technique and sample size

Participants were selected using a purposive sampling approach during the study period. A total of 102 children diagnosed with complicated malaria met the eligibility criteria and were included in the analysis. The sample size was based on the number of eligible cases presenting to the hospital during the study duration and was considered sufficient to examine associations between biochemical parameters and clinical outcomes in this population.

Data collection

Clinical information, including detailed medical history and findings from physical examination, was documented for each participant using a structured data collection form. Patients were categorized into favorable and unfavorable outcome groups. A favorable outcome was defined as complete clinical recovery without complications, whereas an unfavorable/adverse outcome included the development of severe malaria manifestations, organ dysfunction, requirement of intensive care, or death. Laboratory investigations performed for this investigation included measurement of SUA, serum glutamic oxaloacetic transaminase (SGOT), serum glutamic pyruvic transaminase (SGPT), total serum bilirubin, and CRP. SUA concentration was determined using the Uricase-Trinder enzymatic method with an automated biochemical analyzer.

Statistical analysis

All collected data were entered and analyzed using IBM SPSS Statistics for Windows, Version 23 (Released 2015; IBM Corp., Armonk, New York, United States). Continuous variables were summarized as mean ± standard deviation (SD), while categorical variables were expressed as frequencies and percentages. The chi-square test was applied to evaluate associations between categorical variables. The relationship between SUA levels and other liver function indicators was evaluated using Spearman's rank correlation analysis. A p-value of less than 0.05 was considered statistically significant.

## Results

Demographic characteristics

A total of 102 children with complicated malaria were included in the study. The socio-demographic attributes of the research participants are shown in Table 1. The mean age of the study participants was 7.25 ± 3.9 years, with an age range of 1.5-18 years. More than half of the participants (51.0%) belonged to the 1-6 years of age group, then 7-12 years (33.3%), and 13-18 years (15.7%). Female participants constituted 54.9% of the study population, while 45.1% were male. The distribution of participants according to place of residence showed that 50% belonged to rural areas and 50% to urban areas.

**Table 1:** Socio-demographic characteristics of study participants (n = 102)

Variable	Category	Frequency (n)	Percentage (%)
Age group (years)	1–6	52	51.0
	7–12	34	33.3
	13–18	16	15.7
Gender	Male	46	45.1
	Female	56	54.9
Place of residence	Rural	51	50.0
	Urban	51	50.0

Laboratory abnormalities observed among the research participants are summarized in Table 2. Elevated SUA levels (measured in mg/dl) were observed in 80.4% of participants, while 19.6% had values within the normal range. Abnormal liver function markers were also frequently observed. Elevated SGOT levels were present in 72.5%, SGPT in 83.3%, and total serum bilirubin in 65.7% of participants. Additionally, 71.6% of participants had positive CRP, indicating a systemic inflammatory response.

**Table 2:** Distribution of laboratory abnormalities among study participants (n = 102) SUA: Serum uric acid; SGOT: serum glutamic oxaloacetic transaminase; SGPT: serum glutamic pyruvic transaminase

Laboratory Parameter	Normal n (%)	Abnormal n (%)	Reference Range
SUA (mg/dL)	20 (19.6)	82 (80.4)	2.0 – 5.5
SGOT (U/L)	28 (27.5)	74 (72.5)	15 – 40
SGPT (U/L)	17 (16.7)	85 (83.3)	10 – 40
Total serum bilirubin (mg/dL)	35 (34.3)	67 (65.7)	0.2 – 1.0
CRP (mg/dL)	29 (28.4)	73 (71.6)	< 1

The distribution of clinical complications is shown in Table 3. The most common complication was anemia, which was present in 81.4% of participants. Other complications included jaundice (46.1%), respiratory distress (35.3%), seizures (34.3%), shock (23.5%), and hypoglycemia (21.6%). Less common complications included coma (11.8%), hematuria (8.8%), and refractory shock (5.9%).

**Table 3:** Clinical complications observed among research participants (n = 102)

Complication	Present n (%)	Absent n (%)
Anemia	83 (81.4)	19 (18.6)
Seizure	35 (34.3)	67 (65.7)
Respiratory distress	36 (35.3)	66 (64.7)
Jaundice	47 (46.1)	55 (53.9)
Shock	24 (23.5)	78 (76.5)
Coma	12 (11.8)	90 (88.2)
Hypoglycemia	22 (21.6)	80 (78.4)
Hematuria	9 (8.8)	93 (91.2)
Refractory shock	6 (5.9)	96 (94.1)

A majority of the participants (70.6%) had a favorable outcome, whereas 29.4% experienced adverse outcomes, including death and discharge against medical advice. The association between laboratory markers and clinical results is shown in Table 4. Abnormal levels of SGOT (χ² = 6.499, p = 0.011), SGPT (χ² = 8.500, p = 0.004), and total serum bilirubin (χ² = 8.300, p = 0.004) were significantly associated with adverse outcomes. However, SUA levels (p = 0.115) and CRP status (p = 0.223) did not show a statistically significant association with outcome.

**Table 4:** Association of laboratory markers with outcome *: Statistically significant at p < 0.05. SGOT: Serum glutamic oxaloacetic transaminase; SGPT: serum glutamic pyruvic transaminase

Marker	Adverse n (%)	Favorable n (%)	χ²	p-value
Serum uric acid			2.489	0.115
Normal	3 (15.0)	17 (85.0)		
Abnormal	27 (32.9)	55 (67.1)		
SGOT			6.499	0.011*
Normal	3 (10.7)	25 (89.3)		
Abnormal	27 (36.5)	47 (63.5)		
SGPT			8.500	0.004*
Normal	0 (0)	17 (100)		
Abnormal	30 (35.3)	55 (64.7)		
Total bilirubin			8.300	0.004*
Normal	4 (11.4)	31 (88.6)		
Abnormal	26 (38.8)	41 (61.2)		
CRP			1.485	0.223
Negative	6 (20.7)	23 (79.3)		
Positive	24 (32.9)	49 (67.1)		

Independent sample t-test analysis comparing SUA levels between adverse and favorable outcome groups across different age categories is presented in Table 5. Among 1-6-year-old participants, the average uric acid levels were considerably elevated in the adverse outcome group compared with the favorable outcome group (8.48 ± 1.9 vs. 6.29 ± 2.0 mg/dL, p = 0.017). Similarly, among participants aged 13-18 years, the uric acid levels were considerably elevated in the adverse outcome group (10.6 ± 4.1 mg/dL) compared with the favorable outcome group (6.52 ± 2.2 mg/dL, p = 0.039). However, no variations were found to be statistically significant in other age groups.

**Table 5:** Independent sample t-test comparing serum uric acid levels and outcome among age groups Serum uric acid (measured in mg/dL unit); *: Statistically significant at p < 0.05.

Age	Number of Patients	Mean Uric Acid (Adverse) n=30	Mean Uric Acid (Favorable) n=72	Mean Difference	t-value	p-value
1–6 years	52	8.48 ± 1.9	6.29 ± 2.0	2.19	5.11	0.017*
7–12 years	34	8.44 ± 2.7	6.94 ± 2.3	1.50	2.67	0.163
13–18 years	16	10.6 ± 4.1	6.52 ± 2.2	4.07	5.14	0.039*

Spearman’s rank correlation analysis was conducted to examine the relationship between SUA levels and other liver function markers (SGOT, SGPT, and total serum bilirubin) among different age groups of the study participants. As summarized in Table 6, weak positive correlations were observed between SUA and liver enzymes in most age groups. However, none of these correlations were statistically significant (p > 0.05), indicating that SUA levels were not significantly associated with liver function abnormalities among children with complicated malaria.

**Table 6:** Spearman’s correlation between serum uric acid and liver function markers SGOT: Serum glutamic oxaloacetic transaminase; SGPT: serum glutamic pyruvic transaminase

Age Group	n	SGOT (r)	p-value	SGPT (r)	p-value	Total Bilirubin (r)	p-value
1–6 years	52	0.300	0.176	0.254	0.254	0.098	0.666
7–12 years	34	0.085	0.656	0.106	0.577	0.016	0.932
13–18 years	16	-0.057	0.806	0.094	0.685	0.252	0.270

## Discussion

The present study assessed SUA levels in children diagnosed with complicated malaria who were admitted to a tertiary care hospital in eastern India. The results indicated that hyperuricemia was highly prevalent among pediatric patients with severe malaria, with elevated SUA detected in more than four-fifths of the participants. These findings suggest that increased uric acid production may be a frequent biochemical alteration in severe malaria and could reflect the heightened inflammatory activity associated with infection.

Severe malaria develops through complex interactions between parasite multiplication and host immune responses. During the erythrocytic phase of infection, repeated cycles of parasite replication result in the destruction of infected RBCs and the release of parasite-derived antigens and metabolic products into the bloodstream. These substances activate innate immune pathways and stimulate the secretion of inflammatory cytokines, which can cause inflammation throughout the body and subsequent organ dysfunction [11,12].

Uric acid has been increasingly recognized as an important contributor to this inflammatory process. As part of parasite metabolism, infected erythrocytes accumulate hypoxanthine, a purine metabolite. When these erythrocytes rupture, hypoxanthine is released into circulation and is rapidly converted into uric acid by the enzyme xanthine oxidase [15]. Elevated uric acid concentrations may enhance immune activation by stimulating inflammatory pathways and promoting cytokine production [17].

Experimental evidence indicates that uric acid crystals can activate the NLRP3 inflammasome, which leads to the release of inflammatory mediators such as interleukin-1β and contributes to systemic inflammatory responses [18]. These mechanisms are believed to play a role in the pathophysiology of severe malaria.

The high proportion of patients with hyperuricemia observed in this study is consistent with previous clinical investigations. Lopera-Mesa et al. reported that plasma uric acid concentrations were significantly higher in children with severe malaria than in those with uncomplicated infection [20]. Other studies have also demonstrated associations between increased uric acid levels and markers of endothelial dysfunction in malaria patients [21,22].

Endothelial activation represents a key mechanism in the development of severe malaria. Parasitized erythrocytes adhere to vascular endothelial cells through parasite-derived adhesion molecules, resulting in microvascular obstruction and impaired tissue perfusion. The release of inflammatory mediators during infection further aggravates endothelial injury and promotes tissue hypoxia [24].

In the present study, abnormalities in liver function tests were also frequently observed. Elevated SGOT and SGPT levels were detected in more than seventy percent of patients. Hepatic involvement in severe malaria has been widely documented and may arise due to hemolysis, sequestration of infected erythrocytes within hepatic microvasculature, and inflammatory injury to hepatocytes mediated by cytokines [25,26]. The increased bilirubin levels observed in our participants further support the presence of hepatic dysfunction in complicated malaria.

Although hyperuricemia was highly prevalent in the study population, SUA levels were not significantly associated with overall clinical outcomes. Nevertheless, higher uric acid concentrations were observed in some patients with unfavorable outcomes in certain age groups. These findings suggest that while elevated uric acid levels may reflect inflammatory activation, they may not independently predict mortality in malaria.

The absence of a strong correlation between uric acid levels and liver function parameters indicates that increased uric acid may primarily reflect systemic inflammatory processes rather than direct hepatic injury. Similar observations have been reported in studies evaluating other biochemical markers of malaria severity [27].

Another notable observation in the present study was the high frequency of anemia among children with complicated malaria. Severe anemia is a well-recognized complication of *Plasmodium falciparum* infection and occurs due to destruction of infected erythrocytes, removal of uninfected erythrocytes by the spleen, and suppression of erythropoiesis [28]. This reduction in red cell mass may worsen tissue hypoxia and contribute to disease severity.

Respiratory distress and seizures were also commonly encountered among the study participants. These clinical manifestations are characteristic features of severe malaria and may arise from metabolic acidosis, cerebral involvement, or systemic inflammatory responses associated with infection [29]. Early identification and prompt treatment of these complications are essential for improving survival outcomes in affected children.

Limitations

Certain limitations of this study should be acknowledged. First, the research was conducted in a single tertiary care center, which may limit the generalizability of the findings to other geographic regions or healthcare settings. Second, the relatively small sample size may have reduced the statistical power to detect significant associations between SUA levels and clinical outcomes. Finally, measurements of parasite density and inflammatory cytokine levels were not included, which might have provided additional insights into the relationship between uric acid metabolism and malaria pathogenesis.

## Conclusions

In summary, the findings of this study indicate that hyperuricemia is frequently observed in children with complicated malaria and may reflect the inflammatory processes associated with *Plasmodium falciparum* infection. However, SUA levels alone did not demonstrate a significant association with adverse clinical outcomes. Further large-scale, multicenter studies incorporating additional inflammatory biomarkers are required to clarify the prognostic value of SUA in severe malaria and its potential role in clinical risk assessment.
